# Supplementary material for: Modification of the Linker Amino Acid in the Cell-Penetrating Peptide NickFect55 Leads to Enhanced pDNA Transfection for In Vivo Applications
Source: Pharmaceutics. 2023 Mar 9;15(3):883. doi: 10.3390/pharmaceutics15030883 (PMC10051810; doi:10.3390/pharmaceutics15030883)
Supplement: Supplementary file 1 [file pharmaceutics-15-00883-s001.zip › pharmaceutics-2265060-supplementary.pdf]

Supplementary table S1. Sequence of NF55 analogues, where Dap refers to diaminopropionic acid, Dab refers to diaminobutyric acid, Orn refers to Ornithine, Lys refers to Lysine and C18 refers to stearic acid residue attached to the N-terminus of the peptide.

AA	Name	Sequence	MW	RT[1]	222/208 ratio[2]	HLB[3]	H bond donor[4]	H bond acceptor[4]
**Linear peptides**								
Dap	NF55-Dap	C18-AGYLLG**Dap**INLKALAALAKAIL-NH_2	2349	13.025	0.803	11.65	35.79	48.21
Dab	NF55-Dab	C18-AGYLLG**Dab**INLKALAALAKAIL-NH_2	2363	12.882	0.825	11.58	35.95	48.04
Orn	NF55-Orn	C18-AGYLLG**Orn**INLKALAALAKAIL-NH_2	2377	12.836	0.818	11.52	35.95	48.05
Lys	NF55-Lys	C18-AGYLLG**Lys**INLKALAALAKAIL-NH_2	2391	12.879	0.809	11.45	35.96	48.04
**Kinked peptides**								
Dap*	NF55-Dap*	C18-AGYLLG**Dap***INLKALAALAKAIL-NH_2	2349	12.764	0.863	11.65	35.30	48.70
Dab*	NF55-Dab*	C18-AGYLLG**Dab***INLKALAALAKAIL-NH_2	2363	12.701	0.817	11.58	35.62	48.38
Orn*	NF55	C18-AGYLLG**Orn***INLKALAALAKAIL-NH_2	2377	12.668	0.829	11.52	35.69	48.31
Lys*	NF55-Lys*	C18-AGYLLG**Lys***INLKALAALAKAIL-NH_2	2391	12.524	0.823	11.45	35.80	48.20

* – Synthesis is continued via amino group in the side chain. [1] – Retention times in minutes obtained by UPLC. [2] – 222/208 ratio from CD. [3] – calculated hydrophilic-lipophilic balance, Griffin scale, based on structures of peptides drawn in Marvin. [4] – H bond donor and acceptor calculated at pH 7.0 based on structures of peptides drawn in Marvin. Marvin was used for drawing, displaying and characterizing chemical structures, substructures and reactions, Marvin version 20.11, ChemAxon (https://www.chemaxon.com)

Supplementary table S2. Hydrodynamical diameter of CPP/pDNA complexes. Experiments were performed using a Zetasizer Nano ZS apparatus (Malvern Instruments, UK). Complexes were formed between NF55 analogues and luciferase encoding pDNA (pLuc) at CR3 and diluted 10-fold prior the measurements.

CPP/pDNA	Size[1]	pDI[2]
NF55-Dap	155.4 ± 5.2	0.29 ± 0.06
NF55-Dab	135.8 ± 4.0	0.25 ± 0.02
NF55-Orn	159.8 ± 7.2	0.38 ± 0.05
NF55-Lys	134.5 ± 1.6	0.18 ± 0.01
NF55-Dap*	127.6 ± 9.7	0.37 ± 0.03
NF55-Dab*	136.9 ± 7.1	0.19 ± 0.09
NF55	132.3 ± 14.5	0.26 ± 0.02
NF55-Lys*	138.5 ± 1.8	0.18 ± 0.01

[1] – Hydrodynamical diameter of nanoparticles in nanometers. [2] – Polydispersity index

Supplementary table S3. *In vivo* safety profile of NF55 analogues which were efficient in pDNA delivery *in vivo*: ALAT and ASAT levels measured from the blood of mice and weight of mice in grams before injecting with NF55 analogue/pDNA nanoparticles versus before sacrificing mice for *ex vivo* tissue analysis.

CPP/pDNA	ALAT[1]	ASAT[2]	Pre-injection weight	Post-injection weight
Naked pDNA	183	192	25.41	25.55
NF55-Dap	66	237	27.65	25.99
NF55-Dab*	474	131	26.64	25.65
NF55	629	184	26.96	25.35
NF55-Lys*	88	338	28.03	26.19

[1] – Alanine aminotransferase [2] – Aspartate aminotransferase

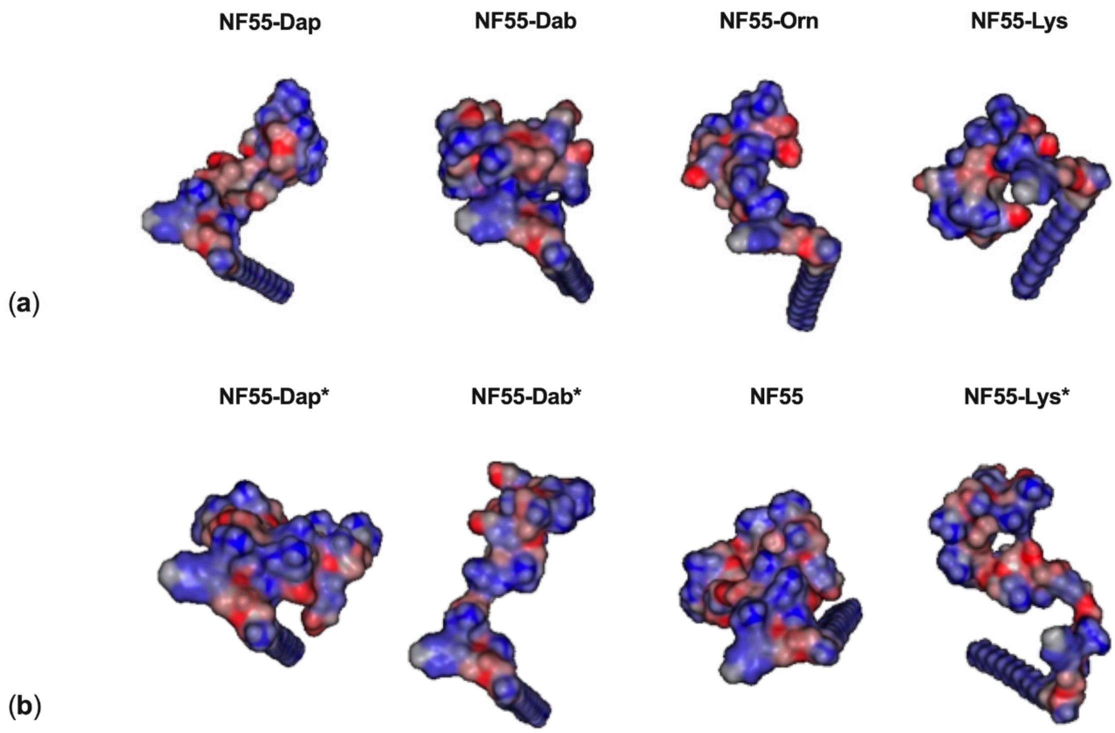

Supplementary Figure S1. 3D projections of (a) linear and (b) kinked NF55 linker analogues.

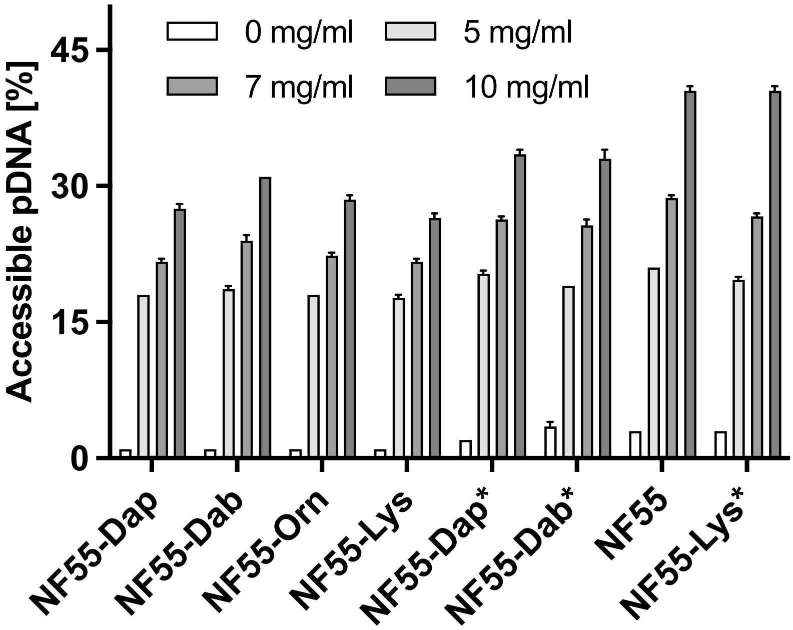

Supplementary Figure S2. Stability of CPP/pDNA complexes against displacement by heparin. Complexes were formed between NF55 analogues and pLuc at CR2. Available pDNA was quantified with NA intercalating dye.

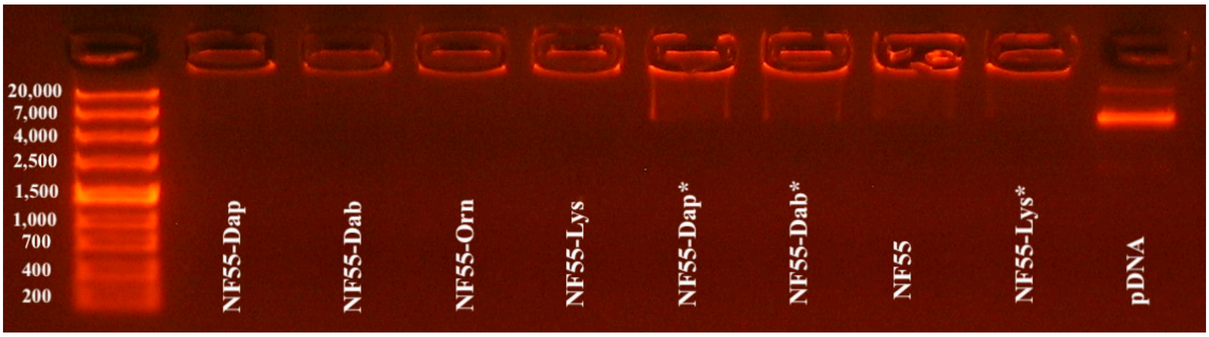

Supplementary Figure S3. Ability of NF55 analogues to interact with pDNA and limit its migration in agarose gel. Complexes were formed between NF55 analogues and pLuc (size 4731 bp) at CR2. After incubation, samples were diluted, loading dye was added and samples were transferred to agarose gel tooth. Gel electrophoresis was performed in agarose gel (1% agarose gel in 1x TAE buffer) in 1x TAE buffer at 80 mA for 1 h. Available pDNA was visualised under UV light using ethidium bromide. Ladder: ZipRuler Express DNA ladder 2

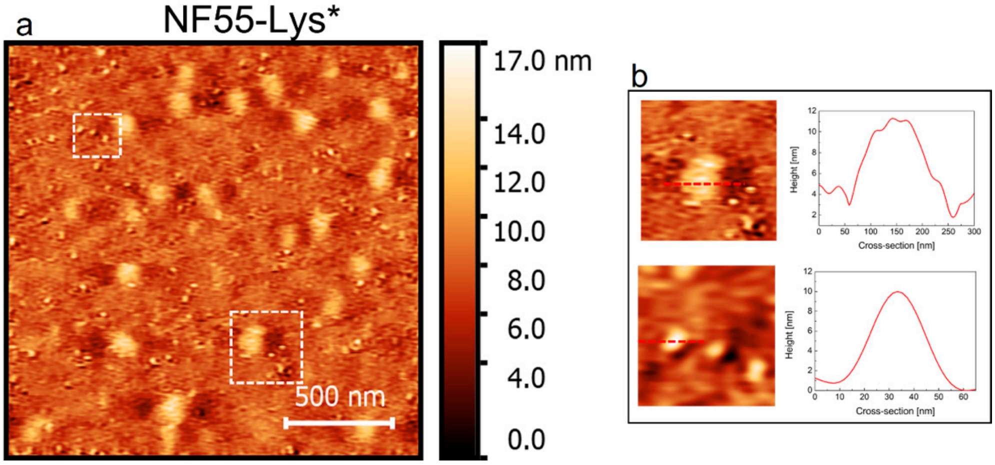

Supplementary Figure S4. AFM-IR data of CPP/pDNA complexes. (a) Representative topographical image of CPP/pDNA complexes, which were prepared here using NF55-Lys*. (b) Profile analyses reveal that the samples contain globular aggregates with sizes in the hundreds of nanometers range. In addition to larger aggregates, smaller complexes are also observed.

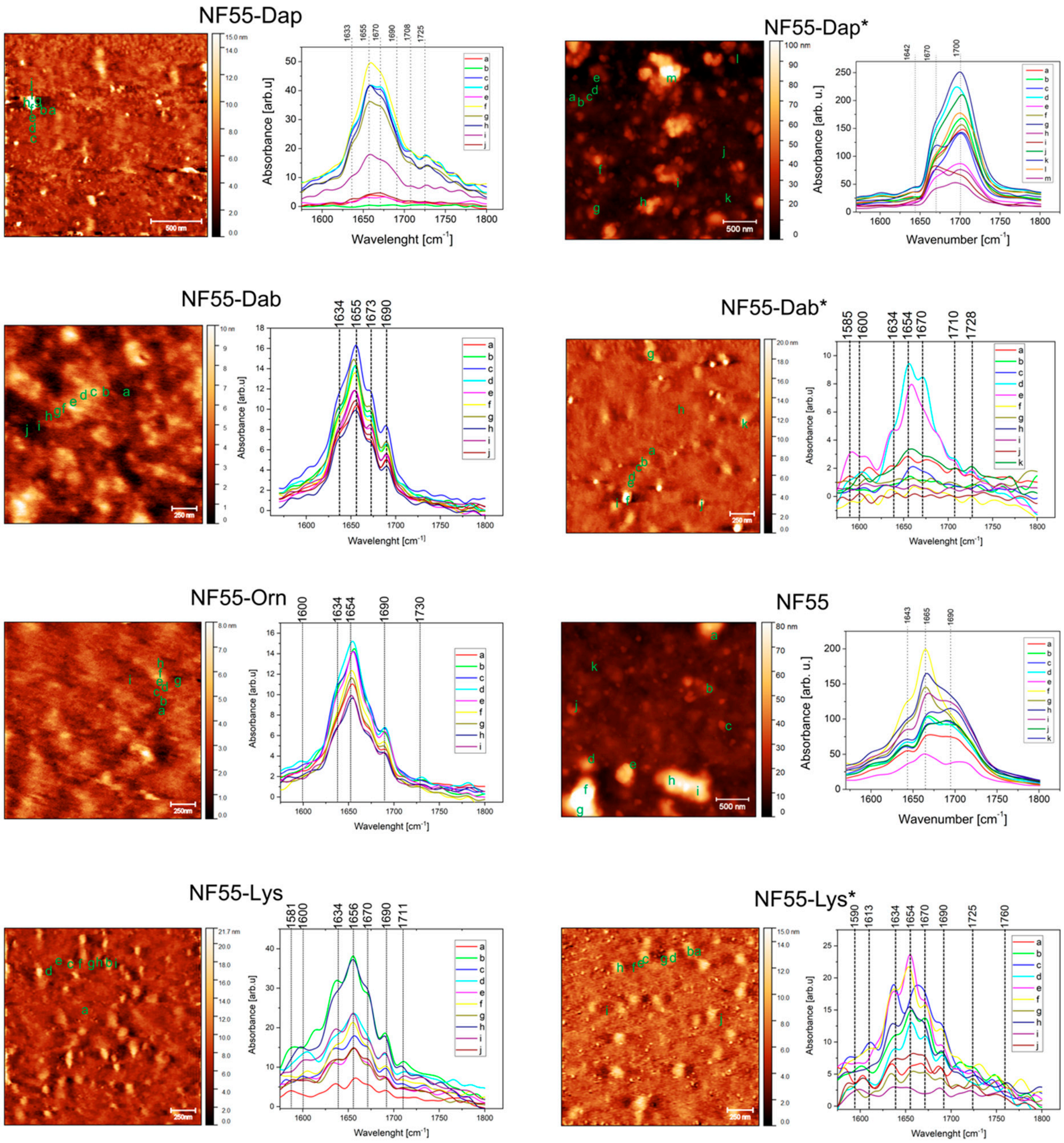

Supplementary Figure S5. AFM-IR data of CPP/pDNA complexes. Topography image (located on the left for each CPP analysed) along with IR spectra (located on the right) showing vibrational signature of the regions indicated with letters a–k in topography image.

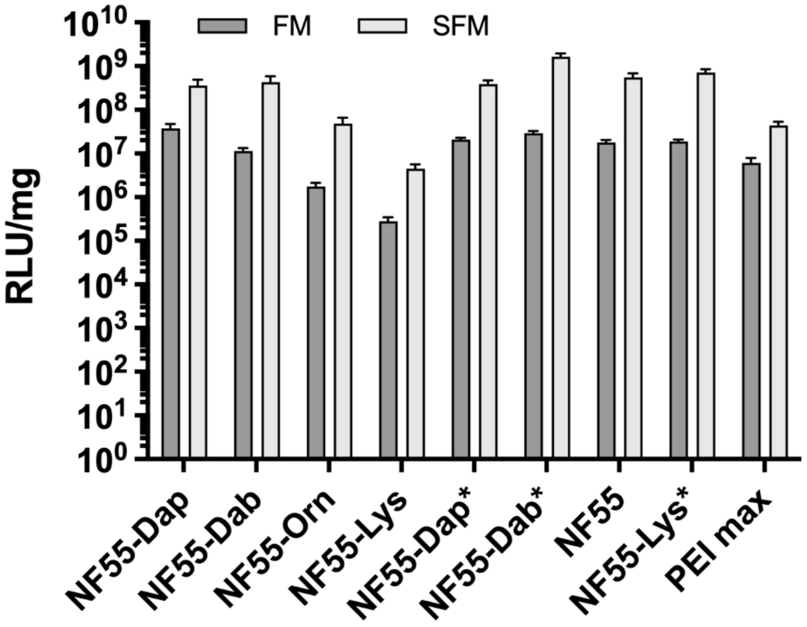

Supplementary Figure S6. Transfection of A549 cells with CPP/pDNA complexes in serum containing media (10% FBS, FM) or in serum free media (SFM). 10,000 A549 cells per well were seeded in a serum containing media 24 hours prior each experiment into 96-well plate. Complexes were formed between NF55 analogues and pLuc at CR3, pLuc dose 0.1 μg per well.

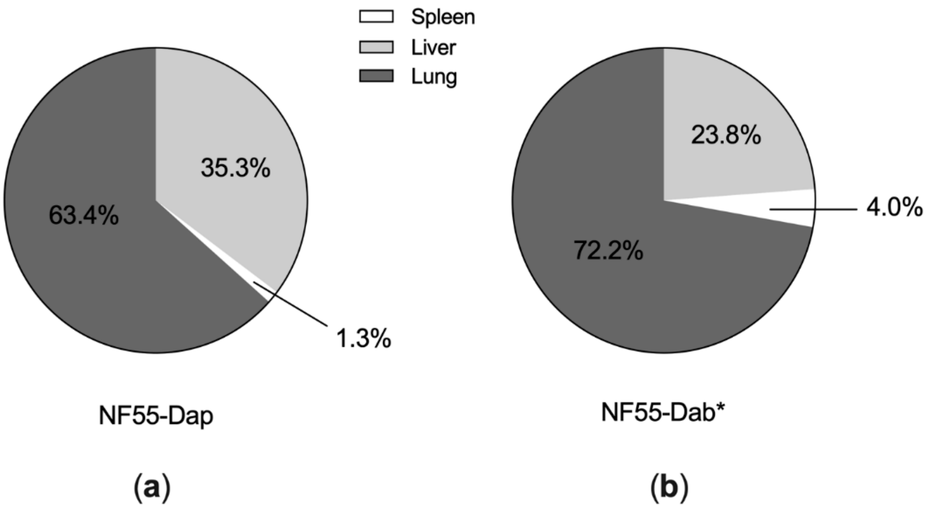

Supplementary Figure S7. (**a**) Distribution of reporter signal between the tissues chosen for analysis of mice injected with NF55-Dap/pDNA complex or (**b**) NF55-Dab*/pDNA complex.
